# Enhanced Exome Sequencing Improves the Genetic Diagnosis of Deafblindness

**DOI:** 10.3390/genes17030344

**Published:** 2026-03-19

**Authors:** Guadalupe A. Cifuentes, Marta Diñeiro, Alicia R. Huete, Raquel Capín, Adrián Santiago, Alberto A. R. Vargas, Dido Carrero, Julien Biscay, Esther López Martínez, Beatriz Aguiar, María Urbaniak, Beatriz Fernández-Vega, María Costales, Rocío González-Aguado, Rubén Cabanillas, Juan Cadiñanos

**Affiliations:** 1 Fundación Centro Médico de Asturias, 33193 Oviedo, Spainalicia.rodriguez@fundacioncma.es (A.R.H.); julien.biscay@fundacioncma.es (J.B.); 2 Instituto de Medicina Oncológica y Molecular de Asturias (IMOMA), 33193 Oviedo, Spainesther.lopez@medastur.com (E.L.M.); ruben.cabanillas@cprecisionc.com (R.C.); 3 Hospital Centro Médico de Asturias, 33193 Oviedo, Spain; beatriz.aguiar@medastur.com (B.A.); maria.urbaniak@medastur.com (M.U.); 4 Instituto Oftalmológico Fernández-Vega, 33012 Oviedo, Spain; beatriz@fernandez-vega.com; 5 Hospital Universitario Central de Asturias, 33011 Oviedo, Spain; 6 Hospital Universitario Marqués de Valdecilla, 39008 Santander, Spain; 7 Cabanillas Precision Consulting, 8020 Zürich, Switzerland

**Keywords:** whole-exome sequencing, panel sequencing, virtual gene panels, non-coding variants, deafblindness, genetic diagnosis, candidate gene discovery

## Abstract

Background/Objectives: The combination of hearing loss and visual impairment in a single patient strongly suggests a genetic aetiology. However, after conventional testing, a considerable proportion of deafblindness cases remain without a genetic diagnosis. The aim of this study was to address this diagnostic gap. Methods: We developed an enhanced exome strategy that uses a whole-exome backbone complemented by spike-in capture probes for (i) low-coverage coding segments and clinically validated, non-coding regions (including deep intronic splice-altering sites and untranslated exonic sequences) across 659 genes associated with hearing loss and/or visual impairment, and (ii) mitochondrial DNA. Results: With 66.6 million paired-end reads per sample, this methodology achieved coverage of at least 20 reads per base at 99.3% of target coding and non-coding positions of genes associated with deafness and/or blindness, as well as 98.8% of the whole exome. The enhanced exome approach correctly identified the genetic variants causative of deafness and/or blindness in 10 out of 10 cases with a previously known genetic cause, in 3 out of 10 additional cases that remained undiagnosed after extensive panel sequencing, and in 4 out of 4 cases that had not been genetically studied before. Comparison of the performance of two commercial bioinformatics platforms for enhanced exome interpretation revealed that eVAI consistently prioritised causative variants higher than, or as high as, VarSome Clinical, resulting in a tendency toward shorter interpretation times using the former. Both platforms offered the same diagnostic yield and both failed to correctly call one of the causative variants. Conclusions: In an era where many centres operate exome analysis through virtual panels, enhanced exome sequencing leverages the advantages of whole-exome and custom panel sequencing: it provides panel-like sensitivity for clinically actionable loci, while offering the flexibility to periodically reanalyse data and discover candidate genes.

## 1. Introduction

Deafness, blindness, and deafblindness are sensory impairments that significantly affect individuals’ quality of life and social integration [1]. While environmental, infectious, or traumatic factors can contribute to these conditions, a substantial proportion of cases have a genetic origin. In recent years, genomic diagnostics have transformed the identification of their hereditary causes, enabling more precise, personalised, and preventive medical care, including the possibility of corrective genetic interventions [2].

Genetic diagnosis can be approached through various strategies, including targeted gene panels, whole-exome sequencing (WES), and whole-genome sequencing (WGS). Targeted panels, which analyse a curated set of genes known to be associated with specific disorders, offer a cost-effective and rapid diagnostic tool with high sensitivity in well-characterised cases [3,4,5]. However, their scope is limited to the genes included, potentially missing pathogenic variants in newly discovered or atypical genes.

Whole-exome sequencing, which captures all protein-coding regions of the genome (approximately 1.5% of the total genome), has proven particularly valuable in the study of rare and genetically heterogeneous disorders, such as inherited deafness and blindness [6,7]. WES enables the identification of pathogenic variants in both known and novel genes, expanding the understanding of the genetic architecture of sensory disorders and improving the diagnostic yield in complex cases. However, standard WES may miss deep intronic regulatory variants and other non-coding genetic alterations that have been shown to be relevant for most genetic conditions, including deafness and/or blindness [8,9].

Whole-genome sequencing provides an even more comprehensive view, encompassing coding and non-coding regions, including introns, promoters, and regulatory elements that may contribute to disease pathogenesis. Although WGS is more expensive and analytically demanding, its utility is increasing with the advancement of bioinformatics tools and the continuous growth of clinical variant databases [6,10,11].

The clinical implementation of these genomic technologies has significantly improved diagnostic rates, facilitated genetic counselling, guided therapeutic decisions, and enabled patient inclusion in clinical trials for emerging gene therapies. Furthermore, genomic diagnostics contribute to the discovery of novel disease genes and molecular mechanisms, driving translational research and the development of innovative treatments.

In this work, we develop and test a strategy aimed at providing clinical-grade sequencing of all genes known to be associated with the genetic conditions present in a patient (in this case, deafness and blindness) while, at the same time, able to produce high-quality exome sequencing from the remaining genes of the genome. Operationally, many diagnostic laboratories currently sequence whole exomes and apply phenotype-driven ‘virtual panels’ for first-pass interpretation, because disease-associated gene sets change rapidly and periodic reanalysis increases diagnostic yield. However, standard exome capture underperforms in certain clinically relevant coding regions and typically does not target known pathogenic deep intronic sites. An enhanced exome design addresses these practical gaps by preserving the exome-wide data needed for future reanalysis and discovery, while boosting clinically critical regions to obtain panel-like sensitivity.

## 2. Methods

### 2.1. Patients

**Figure 1:**
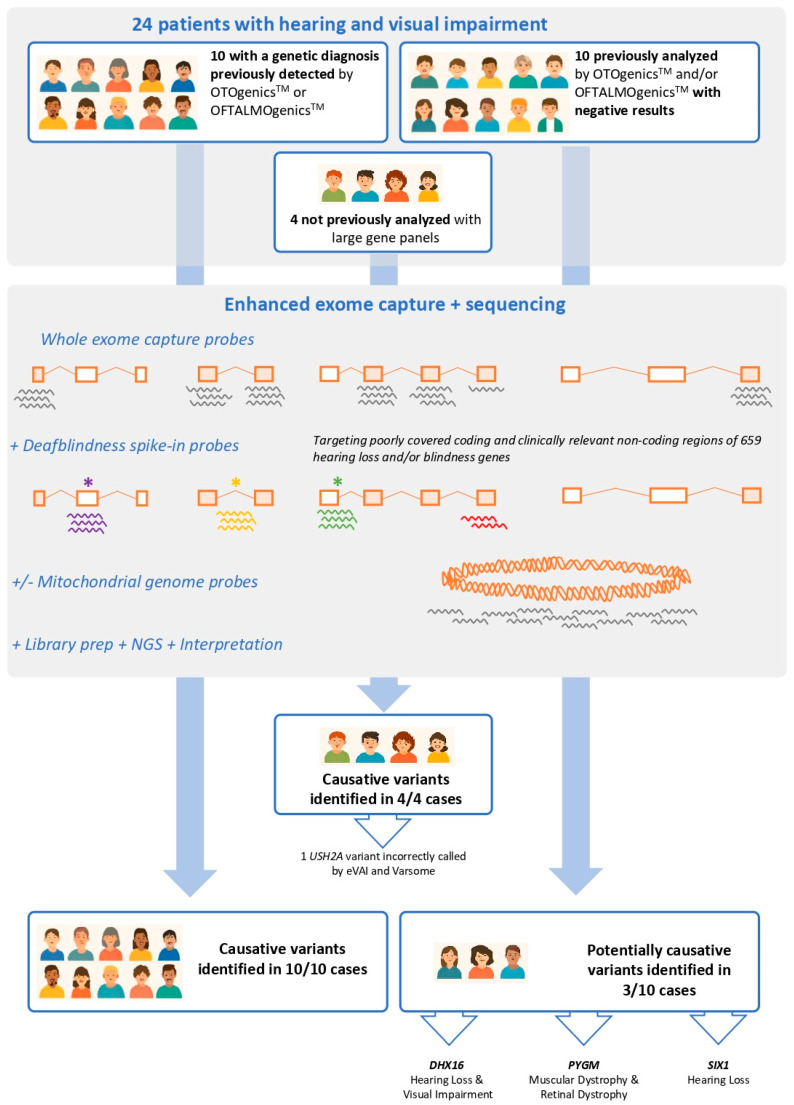
Enhanced exome analysis of 24 deafblind patients. The workflow for enhanced exome sequencing in 24 patients with deafblindness is shown. Top box: Ten patients (#1 to #10) had previously received a genetic diagnosis using OTOgenics™ or OFTALMOgenics™, and their analysis was performed for validation purposes (top left). Ten other patients (#11 to #20) had previously tested negative after being tested with OTOgenics™ or OFTALMOgenics™, so their analysis was performed to evaluate enhanced exome sequencing’s capacity to increase the diagnostic yield (top right). The remaining four patients (#21 to #24) had not previously undergone testing with large gene panels and had no previous genetic diagnoses (top centre). Middle box: Germline DNA samples from all 24 patients were processed using a protocol that involved enrichment of target sequences with probes directed against the whole exome (including the coding exons of all genes) (probes represented as grey ‘coiled’ segments under boxes), supplemented with custom probes designed to capture specific regions of 659 genes associated with hearing loss and/or blindness. These included probes targeted to (1) regions predicted to be poorly covered by the whole exome capture (red probes) and (2) previously described clinically relevant non-coding regions, such as those containing deep intronic splicing variants (yellow asterisk and probes), those within untranslated exons (purple asterisk and probes) or those bearing splice donor sequences of the untranslated exon preceding the first coding exon of each gene (green asterisk and probes). For Patients #13 to #24, the capture included also probes against the whole mitochondrial genome (grey ‘coiled’ segments under mitochondrial chromosome). Bottom: Enhanced exome sequencing found all known causative variants in patients #1 to #10 (bottom left), detected potentially causative variants of hearing loss and/or blindness in three of the patients with previous negative results after panel testing (patients #15, #17 and #19, affected by pathogenic/likely pathogenic variants in *DHX16*, *PYGM* and *SIX1*, respectively) (bottom right) and identified the genetic cause of deafblindness in patients #21 to #24 (including one *USH2A* variant that was not correctly called by eVAI nor VarSome) (bottom middle).

24 patients were involved in this study (Figure 1). 20 of them had been clinically diagnosed with deafblindness and previously tested in our laboratory using the OTOgenics^TM^ and/or OFTALMOgenics^TM^ gene panels [3,4]. Of those, panel testing had previously identified the cause of deafblindness in 10 (included as performance controls), whereas the other 10 remained genetically undiagnosed (included to test the contribution of exome sequencing over panel testing on diagnostic yield). The last four patients were genetically undiagnosed deafblind individuals who had not previously undergone genetic testing. All patients had signed informed consent. This study was approved by the Comité de Ética de la Investigación con medicamentos del Principado de Asturias (CEImPA), with code CEImPA 2022.090.

### 2.2. Samples

19 of the 24 primary samples were peripheral blood preserved in EDTA tubes (4 mL each). Three primary samples were saliva samples preserved in DANASALIVA sample collection kits (Danagen, Badalona, Spain) and 2 of the samples were received as previously extracted genomic DNA. DNA extraction was performed using the FlexiGene DNA Kit (Qiagen, Venlo, The Nethenlands) for blood samples or the DANAGENE Saliva kit (Danagen) for saliva samples.

### 2.3. Enhanced Exome Capture

DNA libraries were constructed using the xGen™ DNA Lib Prep EZ kit (IDT, Coralville, IA, USA), following the manufacturer’s instructions. For enhanced exome capture of the first 12 DNA libraries, we used 500 ng of each library and a mix of 4 μL of the commercially available xGen™ Exome Hyb Panel v2 (IDT) and 1 μL (0.75 pmol) of a custom set of 4161 oligonucleotide probes (Supplementary Table S1) designed to cover the 2640 coding and non-coding target regions (Supplementary File S1) from 659 genes associated with hearing loss and/or blindness (Supplementary Table S2) that were expected to achieve poor coverage according to IDT’s public data (regions containing bases covered by less than 35 reads in at least 2 of 12 samples, when considering 50 million reads per sample) (xGen™ Custom Hyb Panel-Accel; IDT). For capturing the second set of 12 DNA libraries, we used 4 μL of the commercially available xGen™ Exome Hyb Panel v2 (IDT), 0.4 μL (0.3 pmol) of custom probes and 1.6 μL of the xGen™ Human mtDNA Hyb Panel (IDT) probes. We performed 2 capture reactions with 12 samples in each (round 1 with patients #1–12 and round 2 with patients #13–24).

### 2.4. Sequencing

Captured-library sequencing was performed in two runs (12 samples per run) on a NextSeq 550 (Illumina, San Diego, CA, USA), using NextSeq™ 500/550 High Output Reagent Kits (300 cycles) (Illumina).

### 2.5. Coverage Calculations

Before calculating the percentage of positions covered by a minimum of 20 reads (DP20) in each patient, the sequencing results were downsampled to a maximum of 50 million reads per sample in run #1 (25 million from read 1 and 25 million from read 2), and to a maximum of 66.66 million reads per sample in run #2 (33.33 million from read 1 and 33.33 million from read 2). Paired-end FASTQ reads were aligned to the human reference genome (GRCh38.p14, RefSeq GCF_000001405.40) using BWA-MEM2 v2.1.1. SAM alignment files were converted to BAM format, sorted, and indexed using Samtools-1-19-2. Coverage depth per base was calculated within the regions defined in target BED files (Supplementary Files S2 and S3). To estimate the percentage of positions with coverage thresholds (e.g., ≥20× for DP20 or ≥50× for DP50), the total number of positions and those below the threshold were counted from the coverage files. The final percentage of positions meeting or exceeding each coverage level was computed using AWK 1.3.4 scripts.

### 2.6. Identification and Validation of Known and Novel Causative Variants

For the 10 cases with previously known genetic causes of deafblindness (patients #1–10), .bam files generated from enhanced exome sequencing data were visually inspected on IGV to confirm sufficient coverage of the corresponding genomic positions (at least 20 total reads) and the presence and sufficient coverage of each causative variant (at least 10 variant reads). The remaining 14 cases (patients #11–24) were evaluated using eVai (versions: Secondary v1.7, eVAI v3.4; enGenome, Pavia, Italy) and/or VarSome Clinical (version 18.8.2; Saphetor, Lausanne, Switzerland). Pathogenic/likely pathogenic variants considered responsible for deafness and/or blindness were visually confirmed on IGV and further validated by PCR plus Sanger sequencing.

### 2.7. Local Phasing of Nearby Variants

Local phasing of nearby variants was performed using GATK 3.8 ReadBackedPhasing tool, which infers whether variants reside on the same or different haplotypes based on read-level evidence. This step is particularly useful for distinguishing compound heterozygous variants affecting consecutive nucleotides from monoallelic multinucleotide variants.

### 2.8. Comparison of eVAI and VarSome Clinical

To compare the performance of eVAI v3.4 and VarSome Clinical 18.8.2 in identifying variants that cause deafblindness, 10 cases were evaluated in both platforms by an experienced geneticist (tester) who had not been exposed to the cases before. The 10 cases had previously been analysed by a different geneticist (selector). The selector then considered that six cases contained a variant causative of deafness and/or blindness (solved cases), whereas four did not (unsolved cases), and formed two sets of cases (set 1 and set 2) for the tester to analyse, including three solved cases and two unsolved cases in each group. The tester was unaware of the results of the analysis performed by the selector. The selector requested that the tester evaluate each case from set 1 initially with VarSome Clinical and subsequently with eVAI, and each case from set 2 first with eVAI and then with VarSome Clinical. To evaluate the ability, performance, and convenience of eVAI and VarSome Clinical in identifying and prioritising causative variants, the tester recorded the time spent with each case on each platform and the prioritisation order given by each platform to the variants considered causative.

## 3. Results

### 3.1. Content of Deafblindness Enhanced Exome

We gathered a collection of 659 genes associated with nonsyndromic/syndromic sensorineural/mixed hearing loss and/or visual impairment caused by alterations affecting the choroid, vitreous, retina or optic nerve, by combining the updated target gene sets of the OTOgenics^TM^ (v7) [3] and OFTALMOgenics^TM^ (v7) [4] gene panels, including 67 genes associated with deafblindness (Figure 1 and Supplementary Table S2). We then identified non-coding genetic variants that had previously been reported as pathogenic or likely pathogenic by at least one submitter in ClinVar and that affected any of the 659 genes. The resulting list included 165 non-coding variants affecting 63 genes (Supplementary Table S3). We designed 4161 probes (Supplementary Table S1) to capture 2640 genomic regions (Supplementary File S1) containing both those non-coding variant positions as well as target coding regions with the poorest coverage according to public IDT exome research panel v2 sequencing data.

### 3.2. Technical Performance of Enhanced Exome Capture and Sequencing

We conducted an initial round of enhanced exome capture and sequencing, combining the xGen™ Exome Hyb Panel v2 with our custom spike-in probes. Supplementary Table S4 shows the performance of this first round, involving samples from patients #1 to #12 and considering a maximum of 50 million 150-nucleotide-long reads per sample. Our results showed that 98.4% of target positions associated with hearing loss and/or blindness, and 97.4% of whole-exome target positions, were covered by at least 20 reads (Supplementary Table S4 and File S2). To improve these metrics, we performed a second round of enhanced exome capture and sequencing. This involved using a combination of the xGen™ Exome Hyb Panel v2 (IDT), custom spike-in probes and the xGen™ Human mtDNA Hyb Panel during the capture reaction, and considered up to 66 million reads per sample (samples from patients #13 to #24). The second round yielded better results, achieving, on average, at least 20 reads in 99.3% of target bases from regions associated with hearing loss and/or blindness and 98.8% of the whole exome, as well as at least 50 reads in 99.85% of mitochondrial DNA target positions. The % of target bases with 20 or more reads reached 99.72% in positions captured by the spike-in probes, as a consequence of the higher mean coverage (496.76×) obtained in such regions (Table 1 and Supplementary File S3).

### 3.3. Validation of Enhanced Exome Sequencing in Samples with Known Deafblindness-Causing Variants

To check the ability of enhanced exome sequencing to identify the genetic cause of hearing loss and/or blindness, we first sequenced samples from ten deafblind patients (samples #1–10) with known pathogenic or likely pathogenic variants that had been previously detected by OTOgenics™ or OFTALMOgenics™. Enhanced exome sequencing correctly identified the genetic cause of deafblindness in all ten cases (Figure 1 and Table 2; patients #1 to #10).

### 3.4. Diagnostic Yield of Enhanced Exome Sequencing in Samples from Deafblind Patients with Negative Results After OTOgenics™ and/or OFTALMOgenics™ Genetic Testing

To investigate how enhanced exome sequencing can help to close the molecular diagnostic gap in patients with deafblindness who have previously been evaluated with extensive hearing loss and/or blindness genetic panels, we analysed 10 deafblind patients (#11 to #20) that remained genetically undiagnosed after testing with OTOgenics™ and/or OFTALMOgenics™. Enhanced exome sequencing identified potential causes of deafness and/or blindness in three patients: #15, #17 and #19 (Figure 1 and Table 2).

Patient #15, who had been clinically diagnosed with retinitis pigmentosa and severe sensorineural hearing loss, was found to carry the likely pathogenic heterozygous variant *DHX16* (NM_003587.5) c.2021C>T p.(Thr674Met). Pathogenic *DHX16* variants have been associated with neuromuscular disease and ocular or auditory anomalies, sometimes accompanied by seizures (MIM:618733), with autosomal dominant inheritance mode. Specifically, *DHX16* (NM_003587.5) c.2021C>T p.(Thr674Met) has previously been identified as a *de novo* variant in three patients presenting with neuromuscular problems: one of them with developmental delay, central nervous system alterations, myopathy, unspecified eye problems and hearing loss [12]; another with congenital myopathy [13]; and the third one with spasms, neuropathy, myopathy, developmental delay without intellectual disability, and retinopathy [14]. None of the non-ocular or non-auditory manifestations present in these patients were observed in patient #15, which may represent a case of variable expressivity.

In Patient #17, affected by retinitis pigmentosa without pigment, severe hearing loss and muscular dystrophy, enhanced exome sequencing identified a likely pathogenic homozygous variant in *PYGM*: NM_005609.4:c.2392T>C p.(Trp798Arg). *PYGM* pathogenic variants are the genetic cause of McArdle disease (MIM:232600), an autosomal recessive metabolic disorder characterised by the onset of exercise intolerance and muscle cramps during childhood or adolescence. There is a growing body of evidence that links biallelic *PYGM* alterations with pattern dystrophy of the retinal epithelium in patients with McArdle disease, likely caused by higher glucose dependence and uptake in retinal cells [15,16,17,18,19,20]. Interestingly, Patient #17′s ocular phenotype, while not exactly corresponding to pattern dystrophy, resembles that reported by Casalino *et al.* for a patient with macular and mid-peripheral hypofluorescent lesions [20]. Therefore, the identified *PYGM* alteration might explain both the muscular dystrophy and the visual impairment of Patient #17. However, to our knowledge, hearing loss has not yet been associated with *PYGM* alterations or McArdle disease, so it likely originates from a different cause in this patient.

Patient #19 showed macular atrophy, bilateral optic atrophy and sensorineural hearing loss, and enhanced exome sequencing detected in him the heterozygous likely pathogenic *SIX1* variant (NM_005982.4) c.157_160del, p. (Lys53Argfs*35). Disease-causing variants in *SIX1* have been associated with deafness, autosomal dominant 23 (DFNA23; MIM:605192) and branchiootic syndrome 3 (BOS3; MIM:608389). Patient #19 did not show any branchial arch defects, so DFNA23 is considered the most likely genetic diagnosis. This *SIX1* variant, however, did not explain the patient’s ocular phenotype.

### 3.5. Performance of Enhanced Exome Sequencing in Samples from Four Deafblind Patients with No Previous Comprehensive Genetic Testing

Finally, we investigated the potential of enhanced exome sequencing to identify the genetic causes of hearing and/or vision loss in four patients (Patients #21 to #24) who had not previously undergone comprehensive genetic testing (here defined as that involving 10 or more genes). Enhanced exome sequencing detected the genetic cause of deafblindness in all these four cases.

Patient #21, who was born with congenital deafness and began experiencing visual disturbances in his mid-teens, was clinically diagnosed with Usher syndrome at the age of 19 years. Enhanced exome sequencing revealed that he was homozygous for a pathogenic truncating variant in *PCDH15* ((NM_033056.4): c.1737C>G, p.Tyr579*), associated with Usher syndrome, type 1 (MIM:602083).

Patient #22 had atypical retinitis pigmentosa, peripheral vision loss, strabismus, and bilateral cataracts (operated on at the age of 14), as well as progressive hearing loss. She was clinically diagnosed with Usher syndrome when she was 32 years old. However, her syndromic features extended beyond deafblindness, to include trembling limbs and difficulty walking, for which she required the use of a wheelchair. Enhanced exome sequencing found two compound heterozygous pathogenic variants in *ABHD12* (NM_001042472.3: c.846_852dupGCTCTTA, p.(His285*) and c.867+1G>T), associated with polyneuropathy, hearing loss, ataxia, retinitis pigmentosa, and cataract (PHARC) (MIM:612674).

Patient #23 showed signs of cochlear degeneration at 18 months of age and had to use hearing aids since the age of six. Eleven years later, at the age of 17, he experienced retinal detachment, which led to a diagnosis of retinitis pigmentosa. He was also operated on for cataracts. Enhanced exome sequencing identified a homozygous pathogenic truncating variant in *USH2A* ((NM_206933.4): c.9304C>T, p.(Gln3102*)), associated with Usher syndrome, type 2A (MIM:276901).

The last patient of this series, Patient #24, was diagnosed with sensorineural hearing loss in childhood—corrected with hearing aids since the age of seven—and later developed retinitis pigmentosa at the age of 18 years. Enhanced exome sequencing revealed that he was a compound heterozygote for one pathogenic and one likely pathogenic *USH2A* variants (NM_206933.4: c.9304C>T, p.(Gln3102*) and c.1939_1940delinsCC, p.(Gly647Pro), respectively). Of note, the latter variant was not correctly identified by VarSome Clinical or eVAI, which instead reported two separate substitutions (c.1939G>C and c.1940G>C) within codon 647, predicting p.(Gly647Ala) and p.(Gly647Arg), both in the heterozygous state. Manual inspection of sequence alignments confirmed that the substitutions at positions c.1939 and c.1940 occurred on the same allele. Interestingly, a local pipeline using GATK 3.8 ReadBackedPhasing could successfully detect this multinucleotide variant as *USH2A* c.1939_1940delinsCC, p.(Gly647Pro).

### 3.6. Comparison of VarSome Clinical and eVAI for the Interpretation of Enhanced Exome Sequencing Results in Deafblindness

**Figure 2:**
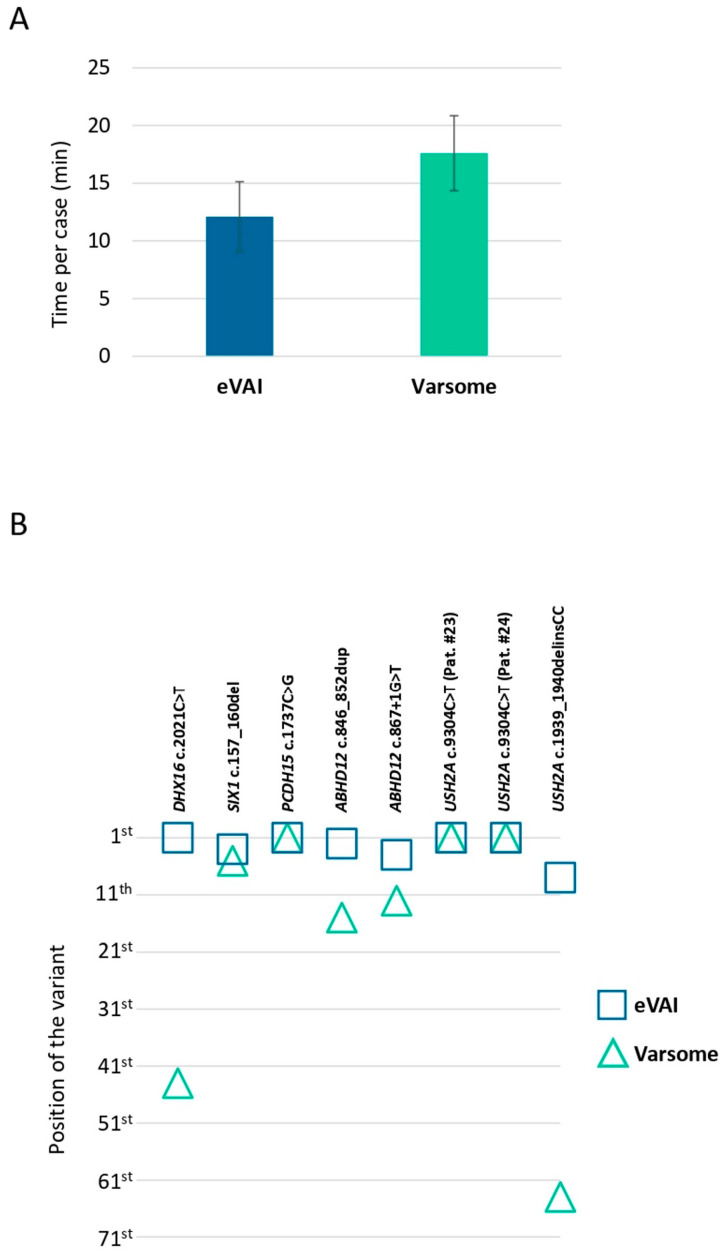
Comparison of eVAI and VarSome in the clinical interpretation of enhanced exome results in deafblind patients. (**A**) The average time spent by an experienced geneticist per case (*n* = 10) on enhanced exome interpretation using eVAI v3.4 (blue bar) or VarSome Clinical 18.8.2 (green bar). See the Section 2.8 for details. Error bars represent the standard error of the mean. (**B**) Prioritisation positions of variants considered causative of deafness and/or blindness in cases analysed in parallel with eVAI v3.4 (blue squares) and VarSome Clinical 18.8.2 (green triangles). *USH2A* c.1939_1940delinsCC p.Gly647Pro was incorrectly identified as c.1939G>C and c.1940G>C by both eVAI and VarSome. Therefore, the position of the highest-ranked variant among the latter two is considered (see Section 2.8 and Supplementary Table S5 for details).

In order to test and select a suitable platform for enhanced exome sequencing interpretation, we compared the performance of eVAI v3.4 and VarSome Clinical 18.8.2. The test involved ten cases from our series, which were analysed by an experienced geneticist (tester) who had not been exposed to them before. The tester analysed five cases (Patients #14, #15, #16, #21 and #22) on VarSome first, then on eVAI, and the other five cases (Patients #18, #19, #20, #23 and #24) on eVAI first, then on VarSome. Another geneticist (selector) had previously chosen these two sets of five cases so that each contained three cases with identifiable deafness and/or blindness causative variants (Patients #15, #19, #21, #22, #23 and #24), and two cases without them (Patients #14, #16, #18 and #20). Although this analysis lacked statistical power, the results of the test revealed a non-significant tendency towards shorter interpretation times on eVAI than on VarSome (12.1 min per case vs. 17.6 min per case, respectively; p-value = 0.232) (Figure 2A). eVAI also appeared to be more effective at prioritising causative variants. Thus, both platforms ranked three causative variants in first position in the corresponding variant lists, whereas for the other variants, eVAI prioritised them better than VarSome in all five cases (Figure 2B and Supplementary Table S5).

## 4. Discussion

Having a timely genetic diagnosis can make a significant difference to the quality of life of patients with hearing loss or visual impairment and, especially, those who suffer from the extreme combination of both, deafblindness. Therefore, those responsible for identifying the genetic causes of these highly disabling conditions should use cost-effective approaches that maximise the likelihood of success.

Although hundreds of genes that cause deafness and/or blindness have been identified, a significant proportion of patients still receive no genetic diagnosis despite undergoing extensive testing, and dozens of novel candidate genes are described every year. Thus, while gene panel sequencing is a valuable tool for genetic diagnosis, its limitation to known hearing loss and/or blindness genes makes it of little use for revisiting the results of previous studies, either once a new disease-associated gene has been identified, or for the discovery of novel candidate genes.

Here, we have developed and tested an approach called enhanced exome sequencing that combines the advantages of disease-focused design with those of comprehensive genetic testing. This approach is based on exome + mitochondrial genome sequencing, complemented by custom probes targeting non-coding regions that contain previously described pathogenic and/or likely pathogenic variants in 659 genes associated with hearing and/or visual impairment. This tool is designed to be valid for routine genetic diagnosis, periodic review of past negative results and novel candidate gene discovery.

One could argue that, compared to whole-genome sequencing, exome sequencing is a suboptimal methodology for identifying novel disease-associated candidate genes. However, candidate genes for genetic conditions are frequently proposed after clearly deleterious alterations (such as those causing premature translation termination or disruption of the canonical intron/exon boundaries) or recurrent/hotspot missense variants have been found in multiple patients that share overlapping disease-associated phenotypes. The information captured by exome sequencing (containing all coding plus splice donor/acceptor sequences) is thus adequate for novel disease-associated discovery, whereas subtler gene alterations, such as deeply intronic variants, are more frequently discovered after the gene has already been proposed as a novel candidate. As for diagnostic purposes, the custom probes targeted against non-coding regions containing previously described pathogenic/likely pathogenic variants make enhanced exome sequencing a valuable tool.

To test these theoretical advantages of enhanced exome sequencing, we applied our approach to sequence the germline DNA of 24 patients with deafblindness. Ten of these patients (Patients #1 to #10) were known to carry pathogenic or likely pathogenic variants that had previously been detected using the OTOgenics™ and/or OFTALMOgenics™ gene panels. Enhanced exome sequencing correctly identified the causative variants in all ten patients. Moreover, in patients #11 to #20, who had previously tested negative for variants using the OTOgenics™ and/or OFTALMOgenics™ panels, our approach detected potential causative variants for deafness and/or blindness in three cases (Patients #15, #17 and #19). Finally, enhanced exome sequencing identified the genetic cause of deafblindness in all four cases that had not undergone comprehensive genetic testing before (Patients #21 to #24).

Considering Patients #15, #17 and #19, the reasons for the lack of genetic diagnoses in these patients with previously negative panel test results varied from case to case, demonstrating the advantages of enhanced exome sequencing over panel sequencing in different situations:

• Patient #15 was found to carry a heterozygous likely pathogenic variant in *DHX16*, a gene currently associated with neuromuscular disease and ocular or auditory anomalies with or without seizures according to OMIM (MIM:618733). Although Patient #15 had been previously tested with the OTOgenics^TM^ and OFTALMOgenics^TM^ panels, none of these included *DHX16* as a target gene. This gene had gone under the radar of the systematic literature review and panel updating protocols in place. However, the availability of exome sequencing data allowed for the detection of this potentially causative variant. Of note, the phenotype of Patient #15 (retinitis pigmentosa and severe sensorineural hearing loss) does not include neuromuscular manifestations, so this could be a case of variable expressivity of *DHX16* (NM_003587.5) c.2021C>T p.(Thr674Met). This variant has been previously detected as a *de novo* variant in three patients with neuromuscular alterations with or without ocular and/or ear defects [12,13,14].
• Patient #17 (with retinitis pigmentosa without pigment, severe hearing loss, and muscular dystrophy) was homozygous for a likely pathogenic variant in *PYGM*, associated with a recessive condition known as McArdle disease (MIM:232600). The OMIM description of this syndrome includes exercise intolerance and muscle cramps, with myoglobinuria that may lead to renal failure, with no reference to vision and/or auditory impairments. Patient #17 had been previously tested by OFTALMOgenics^TM^, which did not contain *PYGM* as a target gene, as its associated phenotype was not considered to overlap with the scope of the panel. However, a growing body of evidence links biallelic *PYGM* alterations with pattern dystrophy of the retinal epithelium in patients with McArdle disease, likely caused by higher glucose dependence and uptake in retinal cells [15,16,17,18,19,20]. Patient #17′s ocular phenotype, while not exactly corresponding to pattern dystrophy, resembles that reported by Casalino *et al.* for a patient with macular and mid-peripheral hypofluorescent lesions [20]. Thus, *PYGM*: NM_005609.4:c.2392T>C p.(Trp798Arg) might explain both the muscular dystrophy and the retinitis pigmentosa in this case.
• Patient #19 (with macular atrophy, bilateral optic atrophy and sensorineural hearing loss) had previously undergone testing with the OFTALMOgenics™ panel, but not the OTOgenics™ panel. While the OTOgenics™ panel contains *SIX1* as a target gene associated with deafness (autosomal dominant 23, DFNA23; MIM:605192) and branchiootic syndrome 3 (BOS3; MIM:608389), OFTALMOgenics™ does not include it, as ocular manifestations have not yet been attributed to *SIX1*. However, enhanced exome sequencing was able to detect the *SIX1* (NM_005982.4) c.157_160del variant, p. (Lys53Argfs*35), considered the most likely genetic cause of patient #19′s hearing loss.

Although the pathogenic *DHX16* variant might explain the ocular and auditory phenotypes of Patient #15, *PYGM* has not been associated with hearing loss, and *SIX1* has not been linked to blindness. Despite exploring potential dual diagnoses, no other causative variants associated with auditory or ocular phenotypes were found in the sequenced regions of Patients #17 and #19. Thus, these two patients represents cases of partial phenotype resolution that could benefit from complementary genetic testing, such as whole-genome sequencing.

Finally, the four cases of deafblindness without previous comprehensive genetic testing (Patients #21 to #24) illustrate the potency of this methodology as a first approach to routine genetic diagnosis: in all of these cases, enhanced exome sequencing identified the causative variants.

The results obtained in this study support our claim that enhanced exome sequencing combines the advantages of disease-focused design and comprehensive genetic testing. These include the identification of the deeply intronic variant *USH2A* (NM_206933.4): c.7595-2144A>G (Patient #9), which would not have been detected by standard whole-exome sequencing, as well as of variants in genes that had not been included in the original panel designs used to test the patients for diverse reasons, but which could be identified thanks to the availability of exome data. Examples of these genes are *DHX16* in Patient #15 and *PYGM* in Patient #17.

Of note, while eVAI seemed to represent a better aid than VarSome Clinical in terms of both time spent for enhanced exome result interpretation and variant prioritisation, none of these commercial platforms were able to correctly identify one of the causative variants in Patient #24: *USH2A* (NM_206933.4):c.1939_1940delinsCC, p.Gly647Pro. This is because this is a multinucleotide variant that cannot be automatically distinguished from two compound heterozygous missense variants affecting two consecutive nucleotides, unless a specific step (such as processing with the GATK ReadBackedPhasing tool) is incorporated into the bioinformatics pipeline. We have informed the local representatives of eVAI and VarSome Clinical of this limitation, which could result in false negatives and false positives in genetic analyses performed using the platform’s current or previous versions, so that they can take this into account when designing and validating future versions of their platforms.

The strategy delineated and validated in this study may be extrapolated to other pathologies. In this context, laboratories focusing on specific disease areas could implement it by incorporating probes targeting the non-coding regions of their genes of interest. Furthermore, the approach could be expanded to all inheritable disease-associated genes in the exome, integrating probes for all pathogenic or likely pathogenic non-coding variants catalogued in ClinVar and other databases of clinically relevant genomic variation. Such an extension would combine the cost-effectiveness and diagnostic immediacy of targeted sequencing with the broader scope of non-targeted genetic analyses, thereby facilitating both the re-evaluation of negative cases and the identification of novel genes involved in human disease.

## 5. Conclusions

Enhanced exome sequencing strikes a practical balance between standard exome sequencing and disease-specific panels for genetically heterogeneous disorders such as deafblindness. By boosting poorly covered coding regions and clinically validated non-coding sites in a curated set of disease-associated genes, while retaining exome-wide coverage, diagnostic sensitivity is increased without compromising reanalysis and discovery potential. We recommend this approach as (i) a first-tier diagnostic test for deafblindness for laboratories that already use exome sequencing with virtual panels and (ii) a robust upgrade path for centres that currently rely on large panels but wish to reduce maintenance and improve future-proofing.

## Figures and Tables

**Table 1:** Enhanced exome performance in round 2 patients.

Patient #	Hearing Loss and/or Blindness Genes		Spike-In Regions		Whole Exome		Mitochondrial DNA	
	DP20 (%)	Mean Cov. (x)	DP20 (%)	Mean Cov. (x)	DP20 (%)	Mean Cov. (x)	DP50	Mean Cov. (x)
Patient #13	99.01	99.56	99.66	438.41	98.63	99.41	100.00	139.66
Patient #14	99.35	119.06	99.77	514.24	98.75	93.8	98.34	88.45
Patient #15	99.39	113.8	99.78	492.09	98.77	94.47	100.00	242.72
Patient #16	99.25	114.96	99.57	495.69	98.69	93.68	100.00	201.94
Patient #17	99.32	116.25	99.78	501.49	98.73	96.06	100.00	177.34
Patient #18	99.47	115.86	99.8	506.59	98.67	95.15	100.00	160.49
Patient #19	99.27	116.34	99.72	506.86	98.84	98.29	100.00	135.6
Patient #20	99.36	114.72	99.78	502.71	98.92	95.39	99.89	123.2
Patient #21	99.24	115.42	99.6	502.8	98.89	96.94	100.00	139.92
Patient #22	99.36	115.15	99.69	504.7	98.69	96.61	100.00	136.98
Patient #23	99.37	114.61	99.73	498.78	98.88	94.18	100.00	139.6
Patient #24	99.35	115.5	99.76	496.74	98.87	94.06	100.00	204.54
**All patients (Average)**	**99.31**	**114.27**	**99.72**	**496.76**	**98.78**	**95.67**	**99.85**	**157.54**

Per-patient and all-patient (average) performance of enhanced exome capture and sequencing using 50 million 150 nt long reads per sample. DP20 and DP50 represent the percentage of target positions covered by at least 20 or 50 reads, respectively. Mean Cov.: mean coverage in each region, corresponding to the average read depth per position in that region.

**Table 2:** Deafblindness causative variants in patients analysed by enhanced exomes.

Patient #	Causative Variants	Zygosity	Clinical Classification	OMIM Phenotype; Inheritance Mode	Phenotype Previous to Genetic Analysis	Age at Diagnosis		Gender	Previously Analysed by Panel?	Causative Variant Previously Known?
						Clinical	Genetic			
**1**	*MYO7A* (NM_000260.4): c.397C>T, p.(His133Tyr)	Homozygous	Likely pathogenic	Usher syndrome, type 1B (MIM:276900); AR	Usher Syndrome	31 y.	33 y.	F	Yes	Yes
**2**	*MYO7A* (NM_000260.4): c.6025delG, p.(Ala2009Profs*32)	Homozygous	Pathogenic	Usher syndrome, type 1B (MIM:276900); AR	Usher Syndrome	10 y.	53 y.	M	Yes	Yes
**3**	*PEX1* (NM_000466.3): c.3077T>C, p.(Leu1026Pro)	Compound heterozygous	Likely pathogenic	Heimler syndrome 1; HMLR1 (MIM:234580); AR	Typical retinitis pigmentosa with pigment, optic atrophy, macular oedema, and severe visual field impairment. Hearing loss.	6 y (Usher)	37 y.	M	Yes	Yes
	*PEX1* (NM_000466.3): c.1548delT, p.(Leu517Cysfs*2)	Compound heterozygous	Likely pathogenic							
**4**	*USH2A* (NM_206933.4): c.9304C>T, p.(Gln3102*)	Homozygous	Likely pathogenic	Usher syndrome, type 2A (MIM:276901); AR	Usher Syndrome	30 y.	31 y.	M	Yes	Yes
**5**	*USH2A* (NM_206933.4): c.1724G>A, p.(Cys575Tyr)	Compound heterozygous	Pathogenic	Usher syndrome, type 2A (MIM:276901); AR	Congenital hearing loss (no ophthalmological diagnosis at the time of genetic analysis)	0 y. (hearing loss)	3 y	F	Yes	Yes
	*USH2A* (NM_206933.4): c.1841-2A>G, p.?	Compound heterozygous	Pathogenic							
**6**	*COL4A5* (NM_033380.3): c.3525_3529dupTGGAC, p.P1177Lfs*124	Hemizygous	Likely pathogenic	Alport syndrome (MIM:301050); XL	Alport syndrome. Bilateral sensorineural hearing loss, mild in the right ear and moderate in the left ear, photophobia, blurred vision, right anterior lenticule, and mild right juxtafoveal alteration.	10 y.	12 y.	M	Yes	Yes
**7**	*USH2A* (NM_206933.4): c.1724G>A, p.(Cys575Tyr)	Homozygous	Pathogenic	Usher syndrome, type 2A (MIM:276901); AR	Sensorineural hearing loss (no ophthalmological diagnosis at the time of genetic analysis)	0 y.	15 y.	F	Yes	Yes
**8**	*USH2A* (NM_206933.4): c.11864G>A, p.(Trp3955*)	Homozygous	Likely pathogenic	Usher syndrome, type 2A (MIM:276901); AR	Sensorineural hearing loss (no reported ophthalmological diagnosis at the time of genetic analysis)	<10 y.	46 y.	F	Yes	Yes
**9**	*USH2A* (NM_206933.4): c.7595-2144A>G, p.?	Compound heterozygous	Pathogenic	Usher syndrome, type 2A (MIM:276901); AR/Retinitis pigmentosa 39; RP39 (MIM:613809); AR	Typical retinitis pigmentosa. Post-lingual childhood hearing loss.	30 y (retinitis); 5 y. (hearing loss)	72 y.	F	Yes	Yes
	*USH2A* (NM_206933.4): c.4726_4728del, p.(Lys1576del)	Compound heterozygous	Likely pathogenic							
**10**	*ABHD12* (NM_001042472.3): c.846_852dup, p.(His285*)	Homozygous	Pathogenic	Polyneuropathy, hearing loss, ataxia, retinitis pigmentosa, and cataract; PHARC (MIM:612674)	Severe-profound bilateral hearing loss. Retinitis pigmentosa.	9 y. (hearing loss); 33 y. (nyctalopia + reduced visual field); 38 y. (retinitis)	44 y.	F	Yes	Yes
**15**	*DHX16* (NM_003587.5): c.2021C>T, p.(Thr674Met)	Heterozygous	Likely pathogenic	Neuromuscular oculoauditory syndrome; NMOAS (MIM:618733); AD	Severe sensorineural hearing loss. Retinitis pigmentosa	18 y. (hearing loss); 25 y. (retinitis)	45 y.	F	Yes	No
**17**	*PYGM* (NM_005609.4): c.2392T>C p.(Trp798Arg)	Homozygous	Likely pathogenic	McArdle disease (MIM:232600); AR	Severe hearing loss. Muscular Dystrophy. Retinitis pigmentosa	5 y. (hearing loss); 40 y. (muscular dystrophy); 42 y. (retinitis)	47 y.	F	Yes	No
**19**	*SIX1* (NM_005982.4): c.157_160del, p.(Lys53Argfs*35)	Heterozygous	Likely pathogenic	Deafness, autosomal dominant 23; DFNA23 (MIM:605192), AD/Branchiootic syndrome 3; BOS3 (MIM:608389); AD	Bilateral hearing loss. Macular atrophy	18 y. (hearing loss); 60 y. (macular atrophy)	72 y.	M	Yes	No
**21**	*PCDH15* (NM_033056.4): c.1737C>G, p.(Tyr579*)	Homozygous	Pathogenic	Usher syndrome, type IF; USH1F (MIM:602083); AR	Usher syndrome	19 y.	54 y.	M	No	No
**22**	*ABHD12* (NM_001042472.3): c.846_852dupGCTCTTA, p.(His285*)	Compound heterozygous	Pathogenic	Polyneuropathy, hearing loss, ataxia, retinitis pigmentosa, and cataract; PHARC (MIM:612674); AR	Bilateral cataracts. Usher syndrome. Ataxia	14 y. (cataracts); 32 y. (Usher); 40 y. (ataxia)	75 y.	F	No	No
	*ABHD12* (NM_001042472.3): c.867+1G>T	Compound heterozygous	Pathogenic							
**23**	*USH2A* (NM_206933.4): c.9304C>T, p.(Gln3102*)	Homozygous	Pathogenic	Usher syndrome, type 2A (MIM:276901); AR/Retinitis pigmentosa 39; RP39 (MIM:613809), AR	Bilateral cochlear degeneration. Retinitis pigmentosa	1.5 y. (hearing loss); 17 y. (retinitis)	50 y.	M	No	No
**24**	*USH2A* (NM_206933.4): c.9304C>T, p.(Gln3102*)	Compound heterozygous	Likely pathogenic	Usher syndrome, type 2A (MIM:276901); AR/Retinitis pigmentosa 39; RP39 (MIM:613809); AR	Sensorineural hearing loss. Retinitis pigmentosa	7 y. (hearing loss); 18 y. (retinitis)	33 y.	M	No	No
	*USH2A* (NM_206933.4): c.1939_1940delinsCC, p.Gly647Pro	Compound heterozygous	Likely pathogenic							

Inheritance modes: AD: autosomal dominant; AR: autosomal recessive; XL: X-linked. Gender: F: female; M: male. The two rightmost columns indicate whether the sample had been previously analysed by the OTOgenics/OFTALMOgenics panels and whether a causative variant had been detected before. y.: years.

## Data Availability

The data presented in this study are not publicly available due to privacy/ethical restrictions, but can be obtained on request from the corresponding author.

## Supplementary Materials

The following supporting information can be downloaded at: https://www.mdpi.com/article/10.3390/genes17030344/s1, Table S1: Target genomic coordinates and sequences of spike-in probes; Table S2: Target genes associated with deafness and/or blindness; Table S3: Target intronic/non-coding variants; Table S4: Enhanced exome performance in round 1 samples; Table S5: Prioritisation positions of causative variants responsible for deafness/blindness in ten positive cases analysed in parallel with eVAI and VarSome; File S1: Bed file of the 2640 genomic regions targeted by the probes listed in Supplementary Table S1; File S2: Bed file containing the target regions of round 1 capture; File S3: Bed file containing the target regions of round 2 capture.
